# The SIRT2 Pathway Is Involved in the Antiproliferative Effect of Flavanones in Human Leukemia Monocytic THP-1 Cells

**DOI:** 10.3390/biomedicines10102383

**Published:** 2022-09-24

**Authors:** Caterina Russo, Alessandro Maugeri, Laura De Luca, Rosaria Gitto, Giovanni Enrico Lombardo, Laura Musumeci, Giovambattista De Sarro, Santa Cirmi, Michele Navarra

**Affiliations:** 1 Department of Chemical, Biological, Pharmaceutical and Environmental Sciences, University of Messina, 98166 Messina, Italy; 2 Fondazione “Prof. Antonio Imbesi”, 98123 Messina, Italy; 3 Department of Health Sciences, University “Magna Græcia” of Catanzaro, 88100 Catanzaro, Italy

**Keywords:** flavonoids, SIRT2, naringenin, hesperetin, naringin, neohesperidin, leukemia, cancer, flavanones, citrus

## Abstract

Acute myeloid leukemia (AML) represents the most alarming hematological disease for adults. Several genetic modifications are known to be pivotal in AML; however, SIRT2 over-expression has attracted the scientific community’s attention as an unfavorable prognostic marker. The plant kingdom is a treasure trove of bioactive principles, with flavonoids standing out among the others. On this line, the aim of this study was to investigate the anti-leukemic properties of the main flavanones of *Citrus* spp., exploring the potential implication of SIRT2. Naringenin (NAR), hesperetin (HSP), naringin (NRG), and neohesperidin (NHP) inhibited SIRT2 activity in the isolated recombinant enzyme, and more, the combination between NAR and HSP. In monocytic leukemic THP-1 cells, only NAR and HSP induced antiproliferative effects, altering the cell cycle. These effects may be ascribed to SIRT2 inhibition since these flavonoids reduced its gene expression and hampered the deacetylation of p53, known sirtuin substrate, and contextually modulated the expression of the downstream cell cycle regulators p21 and cyclin E1. Additionally, these two flavanones proved to interact with the SIRT2 inhibitory site, as shown by docking simulations. Our results suggest that both NAR and HSP may act as anti-leukemic agents, alone and in combination, via targeting the SIRT2/p53/p21/cyclin E1 pathway, thus encouraging deeper investigations.

## 1. Introduction

Acute myeloid leukemia (AML) is the most common hematologic neoplasm in adults, characterized by an accumulation of abnormal myeloblasts, most frequently in the bone marrow, leading to its failure and, eventually, death [1]. The incidence of AML is greater for male subjects than female ones, and it was reported to be increased in more developed and economically advanced countries [2]. Despite the acknowledged role of genetic aberrations in the devolvement of AML, its exact etiology still remains elusive [3]. Therefore, scientific community constantly seeks for novel molecular targets to design more effective therapies in counteracting this nefarious disease.

The acetylation state of histones regulates epigenetic processes by affecting transcription factor access to DNA, thus determining gene expression levels. Histone deacetylases (HDACs) are implied in this mechanism, which in turn increases DNA/histone complex compaction [4,5]. Among HDACs, human sirtuins, NAD^+^-dependent enzymes, are the focus of scientific community’s attention owing to the fact that they regulate a large number of cellular pathways implied in cellular aging and age-associated diseases, among which cancer [6]. In particular, SIRT2, one of the seven human sirtuins, is mainly a cytoplasmic enzyme involved in the deacetylation of histones and α-tubulin, as well as many other transcriptional factors (i.e., p53 and NF-κB). Interestingly, elevated mRNA levels of SIRT2 are found in AML patients’ blasts compared to those of healthy subjects. Specifically, it is over-expressed in both intermediate- and poor-risk patients, compared to the favorable-risk ones, as well as being associated with a significantly shorter overall and event-free survival rate. Moreover, SIRT2 over-expression was particularly evident in AML patients belonging to the M5 subtype, according to the French-American-British (FAB) classification of AML, which is defined as acute monocytic leukemia [7]. 

Therefore, compounds able to inhibit SIRT2 activity are thought to be novel therapeutical approaches to ameliorate conditions such as leukemias, among which AML.

Plant kingdom provides an uncountable number of active principles capable of modulating, blocking, or enhancing several cellular pathways, involved in a wide plethora of physio-pathological conditions. Flavonoids stand out among the others for their anti-infective [8], neuroprotective [9,10], antioxidant and anti-inflammatory [11,12,13] and anti-cancer activities [14,15,16,17]. Moreover, it was shown that flavonoids, such as quercetin and derivatives, have already proved their potential in inhibiting SIRT2 [18].

**Figure 1:**
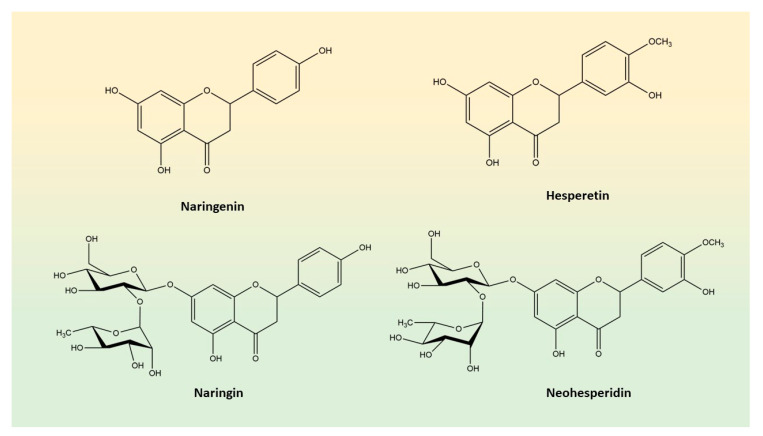
Chemical structures of the main flavanones present in *Citrus* fruits.

On this line, the aim of this study was to investigate whether the main flavanones of *Citrus* spp., namely naringenin (NAR), hesperetin (HSP), naringin (NRG) and neohesperidin (NHP; Figure 1), are able to inhibit the activity of SIRT2 enzyme in both *cell-free* and in vitro models, thus investigating their anti-leukemic activity.

## 2. Materials and Methods

### 2.1. Cell-Free SIRT2 Histone Deacetylase Activity Assay

SIRT2 activity was determined using SIRT2 Direct Fluorescent Screening assay kit (n. 700280) purchased from Cayman Chemical Company (Ann Arbor, MI, USA) [19]. For *cell-free* studies, the histone deacetylase activity was assayed using SIRT2 human recombinant enzyme provided by the kit. Increasing concentrations of HSP, NAR, NRG, and NHP (10, 50, 100, 200, 400 µM; Sigma-Aldrich, Milan, Italy) were tested according to the manufacturer’s guidelines. SIRT2 inhibitor SirReal2 (140 nM; Selleckchem, Houston, TX, USA) was used as positive control. The fluorescence was read after 30 min using a FLUOstar Omega Plate Reader (BMG LABtech, Ortenberg, Germany) at 350–360 nm excitation wavelength and 450–465 nm emission wavelength.

### 2.2. Drug Combination and Analysis of Synergistic Effect

The effects of the combination between increasing concentrations of HSP and NAR (50–400 µM) was assessed in terms of SIRT2 activity on the recombinant isolated enzyme, combined at different ratios, following the checkboard method. In particular, pharmacological interaction models of Loewe additivity and Bliss independence have been developed, and a recent protocol has been employed to measure the Zero Interaction Potency (ZIP) [20]. Results were processed to define interaction between the abovementioned compounds. Synergy scoring was determined using the SynergyFinder 2.0 software [21] that exploits the ZIP calculation method, expressing the synergism as δ score. Positive δ values correspond to synergism, whereas negative ones to antagonism [22].

### 2.3. Cell Culture

The human leukemia monocytic THP-1 cell line was originally obtained from ATCC (Rockville, MD, USA). The cells were grown in RPMI 1640 medium supplemented with 10% *v*/*v* heat-inactivated fetal bovine serum (FBS), L-glutamine (2 mM), HEPES (10 mM), sodium pyruvate (1 mM), glucose (2.5 g/L), 2-mercaptoethanol (0.05 mM), penicillin (100 IU/mL) and streptomycin (100 µg/mL), at 37 °C in a humified 5% CO_2_ atmosphere. Medium was renewed every 2 days and split performed when cells reached maximum density (1 × 10^6^ cells/mL). Human peripheral blood mononuclear cells (PBMCs) were originally obtained from ATCC and cultured using supplemented RPMI 1640 medium (10% *v*/*v* FBS), at 37 °C in a 5% CO_2_ environment. Each reagent for cell culture was from Gibco (Life Technologies, Monza, Italy).

### 2.4. Cell Viability Assays

The evaluation of the antiproliferative activity was assessed by resazurin assay, as an index of mitochondrial functionality, and propidium iodide (PI) staining to detect dead cells [23]. For the former, THP-1 cells and PBMCs were seeded in 96-well plates at a density of 1 × 10^4^ cells/well in 200 µL. Cells were treated with the flavanones NAR, NRG, HSP, NHP (50–400 µM). For vehicle control, we added the same amount of DMSO present in the highest concentration of the flavanones tested to assess whether no toxic effect was induced by the solvent. The plates were then incubated for 24, 48 and 72 h at 37 °C, prior adding 20 µL of resazurin (Santa Cruz, Dallas, TX, USA) 0.01% *w*/*v* solution to each well and kept for further 3 h. Fluorescence was measured on a microplate reader POLARstar Omega (BMG Labtech) with an excitation wavelength of 544 nm and an emission one of 590 nm.

For PI staining, cells (1 × 10^6^ cells/well) were treated with NAR, NRG, HSP, NHP (100–400 µM) in 6-well plates, which were incubated at 37 °C for 72 h. Subsequently, cells were harvested, washed twice and resuspended in 100 µL of PBS plus 10 µL of PI solution (10 µg/mL; Sigma-Aldrich). Stained cells were incubated for 30 min at room temperature in the dark and fluorescence of at least 10,000 events was analyzed by a Novocyte 2000 cytofluorimeter (Agilent, Santa Clara, CA, USA) with FL-2 channel.

### 2.5. Cell Cycle Analysis

The ability of flavanones to interfere with progression of cell cycle was evaluated by flow cytometry [24]. Briefly, THP-1 cells were seeded in 6-well plates (2 × 10^5^ cells/well) and treated with the flavanones NAR and HSP (100–400 µM) or with their combination at concentrations of 100 µM for 24, 48 and 72 h. Then, cells were collected, centrifuged, washed with PBS, and fixed in 70% ice-cold ethanol while gently vortexed. After at least 2 h at 4 °C, cells were centrifugated, washed twice with cold PBS, and resuspended in 250 μL of PBS together with 5 μL of RNase A (10 mg/mL; Sigma-Aldrich) at 37 °C for 1 h. After incubation, 10 µL of PI (1 mg/mL) were added to samples, and immediately acquired by cytofluorimeter. Three independent sets of at least 10,000 events were collected for each condition.

### 2.6. Quantification of Acetylated p53 in THP-1-Treated Cells

To assess the levels of acetylated p53 in THP-1 cells treated for 24 h with NAR and HSP (100, 200 and 400 µM) and their combination (100/100 µM), or with SIRT2 inhibitors SirReal2 (10 µM) and nicotinamide (NAM; 1 mM; Cayman, Ann Arbor, MI, USA) [25], a commercial enzyme-linked immunosorbent assay (ELISA) kit was employed (E4531; Biovision, Milpitas, CA, USA). Briefly, cell lysates from the abovementioned treatments were quantified using Bio-Rad DC Protein Assay (Bio-Rad Laboratory, Hercules, CA, USA) with bovine serum albumin as standard. To equal amounts of protein for each sample, 100 µL of biotin-conjugated primary antibody were added in provided strips and incubated for 1 h at 37 °C. After washing wells, 100 µL of streptavidin HRP-conjugated were added and incubated for additional 30 min at 37 °C. Finally, 90 μL of TMB substrate were added into each well, cover the plate and plate was incubate at 37 °C in dark for further 30 min. Color formation was stopped and absorbance recorded with a microplate spectrophotometer at 450 nm (iMark™ microplate reader, Bio-Rad Laboratories). Results were extrapolated as ratio between values detected in treated and untreated cells.

### 2.7. Real-Time PCR

THP-1 cells were plated in 100 mm Petri dishes at a density of 1 × 10^6^ cells/dish with fresh medium (untreated cells), NAR and HSP (100, 200 and 400 µM), as well as their combination (100/100 µM) at 37 °C for 12 and 24 h. Then, total RNA from untreated and treated cells was extracted using TRIzol reagent (Invitrogen, Carlsbad, CA, USA), according to the manufacturer’s instructions. Afterward, 2 µg of total RNA was reverse transcribed into cDNA using the High-Capacity cDNA Archive Kit (Applied Biosystems, Life Technologies, Foster City, CA, USA), as previously described [26]. Quantitative PCR (qPCR) reactions were performed in triplicate into a 96-well plate, in a final volume of 20 µL, containing 1× SYBR Select Master Mix (Applied Biosystems), 0.2 µM of specific primers and 25 ng of RNA, previously converted into cDNA. The mRNA levels of SIRT2, p53 (TP53), p21 (CDKN1) and cyclin E1 (CCNE1) were analyzed using a 7500 qPCR System (Applied Biosystems), according to the following protocol: one cycle at 95 °C for 10 min, followed by 45 cycles at 95 °C for 15 s, and 60 °C for 1 min. A standard dissociation stage was added to assess the primer specificity. The primer sequences used for qPCR were designed based on those previously published and are listed in Table 1. Data collected were analyzed employing the 2^−∆∆CT^ relative quantification method against β-actin (ACTB), used as the housekeeping control. The values are expressed as *n*-fold change compared to untreated cells. When the value was less than 1, it was converted into its inverse to report downregulated genes.

### 2.8. Docking Studies

Docking studies were performed by AutoDock4 suite [27], using the crystal structure of SIRT2 in complex with SirReal2 retrieved from the RCSB Protein Data Bank (PDB code 4RMG) [25]. The ligand and water molecules were removed, and hydrogens were added by Discovery Studio 2.5. Ligand structures were generated by VEGAZZ suite and optimized by following a conjugate gradient minimization by AMMP calculation, implemented in the VEGAZZ program [28]. Docking simulation was performed by following the same protocol as reported by Roshdy et al. [29], except for the grid center’s coordinates that were defined using the centroid of the co-crystalized ligand SirReal2.

### 2.9. Statistical Analyses

The assays were carried out in different replicates, as described above. Data obtained were expressed as mean ± standard error of the means (SEM). Statistical evaluation of results was performed using one-way analysis of variance (ANOVA), depending on the assay. Multiple comparisons of the means of the groups were performed by the post hoc Student–Newman–Keuls test (SigmaPlot Software, Chicago, IL, USA). The *p*-values lower or equal to 0.05 were considered statistically significant.

## 3. Results

### 3.1. Flavanones Inhibited the Activity of the Isolated Recombinant SIRT2 Enzyme

**Figure 2:**
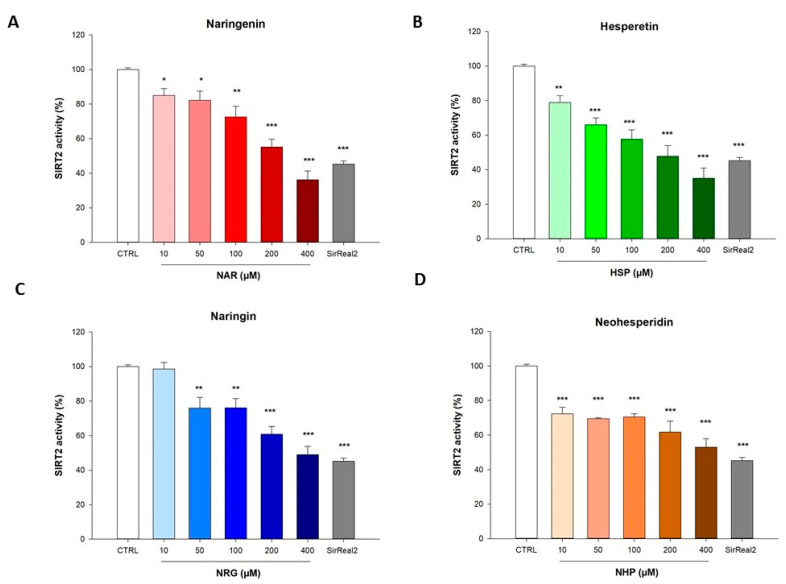
Flavanones inhibited SIRT2 activity in a *cell-free* model. Control (CTRL, white bar) consisted of the enzyme alone, whereas NAR (**A**, red bars), HSP (**B**, green bars), NRG (**C**, blue bars), and NHP (**D**, orange bars) were tested at the concentrations shown in the figure. The SIRT2 inhibitor SirReal2 was employed as standard (gray bar). Data are expressed as percentage of SIRT2 enzymatic activity ± SEM of three independent experiments performed in triplicate (*n* = 9). * *p* < 0.05, ** *p* < 0.01, and *** *p* < 0.001 vs. CTRL.

The inhibition of SIRT2 enzymatic activity induced by the flavanones NAR and HSP, along with their glycosidic counterparts NRG and NHP, respectively, was assayed in a *cell-free* model consisting of the isolated recombinant enzyme. In this setting, each flavanone was able to inhibit SIRT2 activity, despite to a different extent (Figure 2). In detail, at 200 µM, the two aglycones HSP and NAR reduced the enzymatic activity up to 52.3 ± 4.1% and 44.8 ± 3.2%, respectively, whereas, at 400 µM, up to 65± 4.7% and 63.8 ± 3.5%, respectively (Figure 2A,B). On the contrary, NHP and NRG were the weakest among the flavanones tested, reaching an inhibition of 38.2 ± 2.1% and 39.2 ± 4.4% at 200 µM, and up to 47.1 ± 3.2% and 51 ± 4.8% at 400 µM, respectively (Figure 2C,D).

### 3.2. NAR and HSP Acted Synergistically to Inhibit SIRT2 Enzymatic Activity

**Figure 3:**
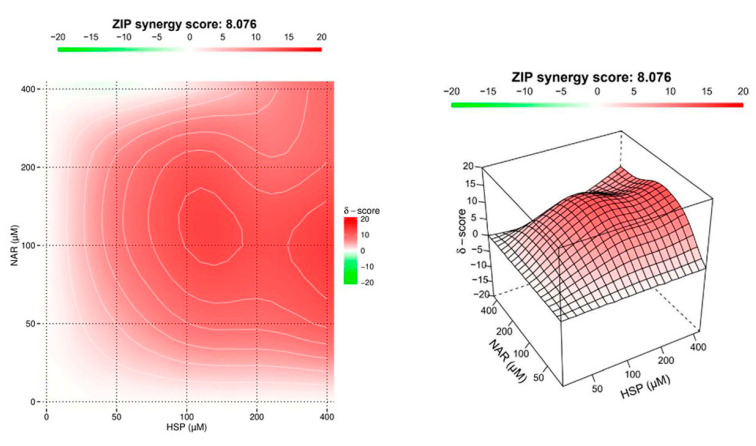
Synergistic effect of the combination between NAR and HSP. The effect of the drug combination on SIRT2 activity was calculated and visualized using SynergyFinder 2.0 software through the ZIP reference model. The synergy score is expressed as the mean of all scores for the dose-response analysis, while the maps shown are representative of three different experimental sessions. The red, white, and green regions of the graphs indicate the synergy, additivity, and antagonism, respectively.

SIRT2 enzymatic activity was further evaluated by testing the combination of single flavanones (NAR, NRG, HSP, and NHP) at different ratios in the isolated enzyme. The obtained results have been processed to investigate the presence of antagonism or synergism. A clear antagonism was observed between the pairs NAR-NHP, NAR-NRG, NHP-NRG, and NRG-HSP, whereas a weak synergistic interaction was detected between NHP and HSP (data not shown). Unlike the other combinations, the flavanones NAR and HSP, which individually elicited the strongest inhibitory effect on SIRT2 enzymatic activity, showed a great synergism in counteracting SIRT2 activity (Figure 3). Indeed, the combination between NAR and HSP displayed an overall sharp synergistic effect with a ZIP score (δ) of 8.076. As showed, the red area (synergism) reaches a peak when the two flavanones are at equimolar concentrations of 100 µM (δ = 12.119). Moreover, the synergism of the two flavanones appears being preserved for equimolar concentrations up to 200 µM (δ = 8.819) and weakens toward higher concentrations. Therefore, the effective combinations of the two flavanones at equimolar concentrations of both 100 and 200 µM were then assessed in vitro.

### 3.3. Flavanones Inhibited THP-1 Cell Proliferation

**Figure 4:**
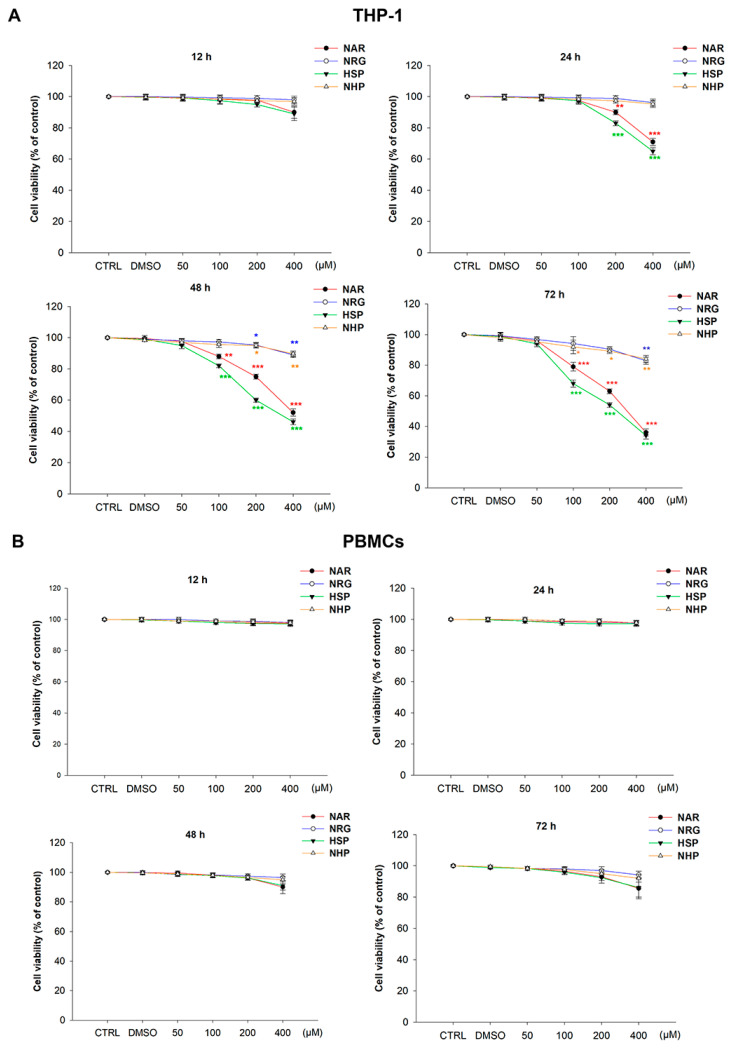
Effects of NAR, NRG, HSP, and NHP (50–400 µM) in THP-1 (**A**) and PBMCs (**B**) cell proliferation. Viability rate was assessed by resazurin assay. Results are expressed as percentages ± SEM of the fluorescence values detected with respect to those of the control (CTRL) cells. Each concentration was tested eight-fold, and three independent experiments were carried out (*n* = 24). * *p* < 0.05, ** *p* < 0.01, and *** *p* < 0.001 vs. CTRL.

To investigate whether the studied flavanones possessed anti-leukemic activity, the effect of NAR and HSP, along with their glycosidic counterparts NRG and NHP, was assessed in a human monocytic leukemia THP-1 cell line, which represents a preclinical model of the M5 subtype of AML. In these cells, both NRG and NHP hampered viability at 72 h by no more than 20% at the highest concentration tested (400 µM). On the contrary, the aglycones NAR and HSP significantly reduced cell proliferation already at 24 h (29.0 ± 2.4% and 35.0 ± 2.2%, respectively) and up to 64.0 ± 2.3% and 66.0 ± 2.4% at 72 h, respectively (Figure 4A).

In order to assess the potential cytotoxicity of the flavanones under study, we repeated the resazurin assay in normal human PBMCs, testing the same timings and concentrations. Noteworthy, none of these caused any inhibitory effects on PBMCs growth, if not after exposure to the flavanones NAR and HSP at the concentration of 400 µM for 72 h, where a slight but not significant reduction in cell viability was observed (Figure 4B).

At this point, we have thus identified non-cytotoxic concentrations employed in the following experiments.

**Figure 5:**
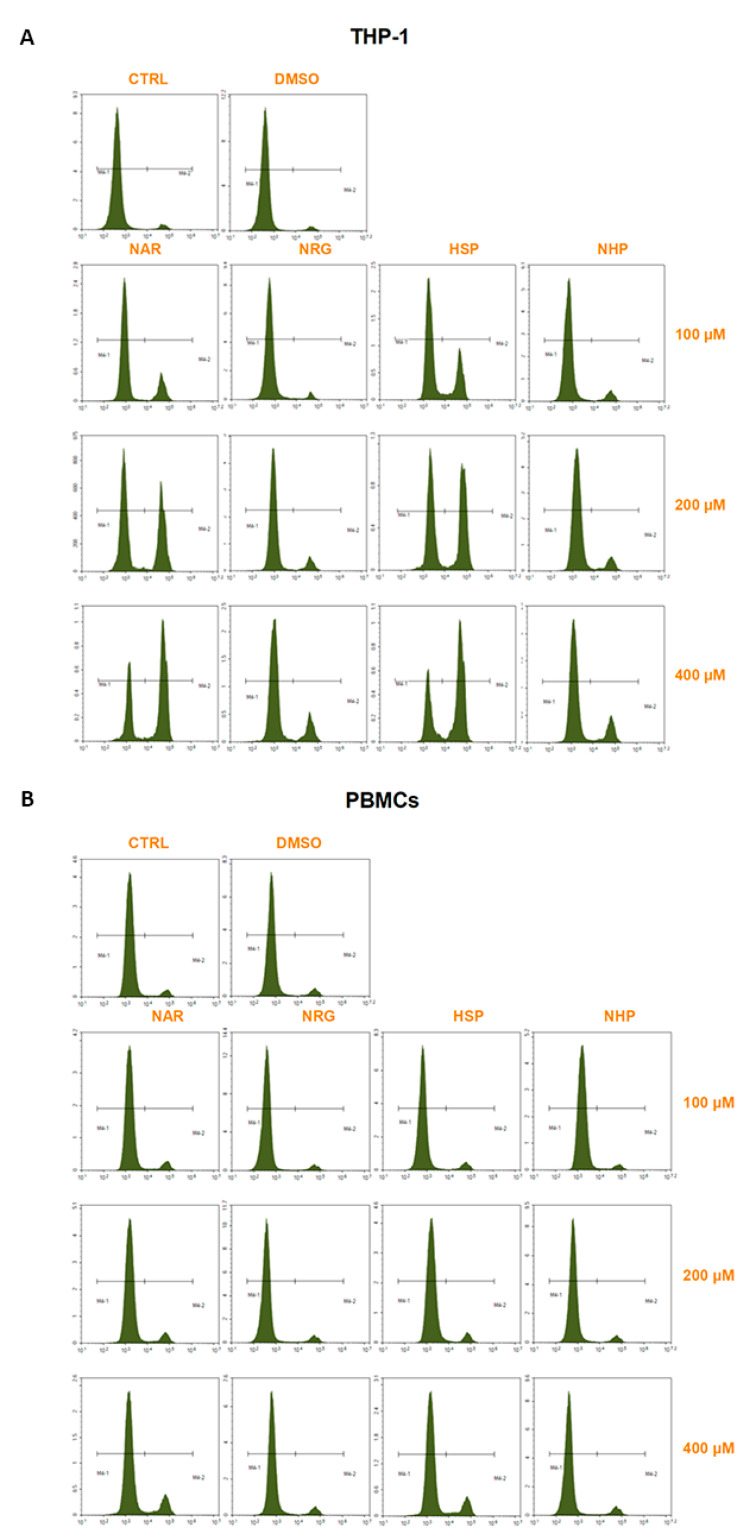
Cytotoxic effects of NAR, NRG, HSP, and NHP (100–400 µM) in THP-1 (**A**) and PBMCs (**B**) cells after 72 h of exposure, assessed by PI staining. Plots are representative of three different experimental sessions of PI staining performed in triplicate (*n* = 9). * *p* < 0.05, ** *p* < 0.01, and *** *p* < 0.001 vs. CTRL.

The PI staining corroborated the outcome observed with the resazurin assay (Figure 5). Consistent with the latter, both NAR and HSP induced cell death in THP-1 monocytes, as demonstrated by the increase in the number of fluorescent cells by 64.5 ± 1.9% and 66.1 ± 3.1% at 72 h, respectively. As for cell viability assay, neither NRG nor NHP was able to induce any damage to the THP-1 cell membrane; thus, no PI diffused up to the nuclear compartment, thus intercalating to DNA (Figure 5A). On the contrary, no flavanone caused significant cytotoxicity against primary cells after 72 h of exposure (Figure 5B).

Given these results, further in vitro experiments were performed considering only NAR and HSP.

### 3.4. NAR and HSP Altered Cell Cycle Progression in THP-1 Cells

**Figure 6:**
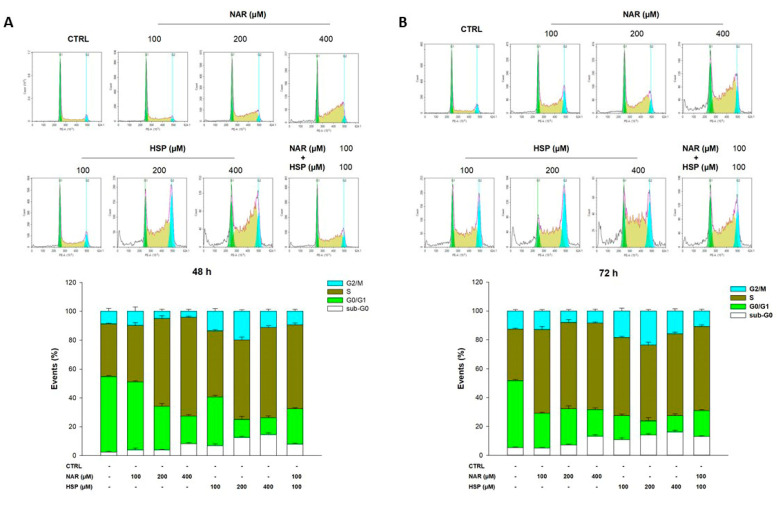
Influence of NAR and HSP, along with their combination, on THP-1 cell cycle progression after 48 h (**A**) and 72 h (**B**) of treatment. The plots are representative of three different experimental sessions of PI staining performed in triplicate (*n* = 9). Percentages of cells present in each phase of the cell cycle are reported in the histograms (sub-G0/G1: white; G0/G1: green; S: yellow; G2/M: cyan).

With the aim of comprehending the mode of death elicited by NAR, HSP, and their combination, their influence on the cell cycle progression of THP-1 cells was evaluated. After 24 h of treatment, just a slight modulation of cell cycle progression was observed with 400 µM of NAR and HSP (data not shown). After 48 h of treatment, 100 µM of NAR did not elicit any modification of the ratio among cell cycle phases respect to the control, whereas 200 µM and even more 400 µM increased the percentage of cells in S phase. Similarly, 100 µM of HSP did not alter the cell cycle at 48 h, while 200 µM increased the cell population in S and G2/M phases. The accumulation of cells in the S phase was also recorded with HSP at 400 µM concentration. Moreover, the combination between NAR and HSP at a molar ratio of 1:1 (100 µM) displayed a stronger effect than the two flavanones alone (Figure 6A). Consistent with the synergistic interaction observed in the abiotic assay (red area of Figure 3), the combination between NAR and HSP at a concentration of 200 µM produced an effect comparable to that from the 100 µM combination (data not shown). After 72 h of treatment, both NAR and HSP at 100 and 200 µM brought a sharp increase in cell population in the S phase, with HSP also in G2/M one. For both flavanones, the S phase arrest is even more appreciable at a concentration of 400 µM (Figure 6B). Remarkably, as for the 48-h treatment, the 100 µM combination between the two flavanones elicited a stronger effect than that recorded for single compounds. A similar block of the cell cycle as that of the 100 µM combination was also experienced with the 200 µM one (data not shown).

Of note, cells populating the sub-G0/G1 phase (hypodiploid cells), a known sign of apoptosis, are relevant after treatment with HSP (all tested concentrations), NAR (400 µM), and their combination (100/100 µM) at both 48 and 72 h (Figure 6).

### 3.5. NAR and HSP Increased Levels of Acetylated p53 in THP-1 Cells

**Figure 7:**
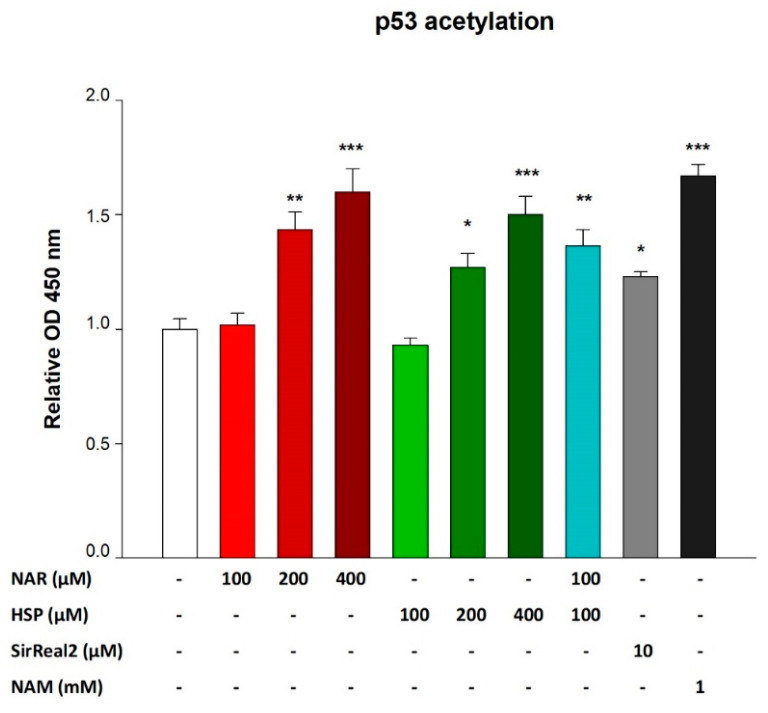
Relative levels of acetylated p53 after treatment for 24 h with NAR (red bars), HSP (green bars), and their combination (light blue bar) in THP-1 cells. Specific and non-specific SIRT2 inhibitors, SirReal2 (gray bar) and NAM (black bar), respectively, were used as positive controls. Protein levels of acetylated p53 were quantified by an ELISA. Data are expressed as fold change with respect to controls (untreated cells) and are the mean ± SEM of three independent experiments performed in triplicate (*n* = 9). * *p* < 0.05, ** *p* < 0.01, and *** *p* < 0.001 vs. CTRL.

As a direct in vitro effect of SIRT2 activity modulation, the levels of acetylated p53 protein were evaluated by means of an ELISA. In detail, treatment with both NAR and HSP 100 µM for 24 h did not alter levels of acetylated p53 with respect to control cells (Figure 7). On the contrary, NAR 200 µM increased acetylation of p53 by 1.43-fold, while HSP at the same concentration by 1.27-fold, with respect to controls. In turn, NAR 400 µM increased p53 acetylation up to 1.64-fold as well as HSP 400 µM to 1.53-fold, compared to controls. Notably, the combination between NAR and HSP (100 µM) elicited an enhancement of the rate of p53 acetylation by 1.37-fold with respect to controls (Figure 7). Similarly, this effect occurred with a combination of 200 µM (data not shown).

Finally, to appropriately compare the abovementioned results, we used a specific (SirReal2, 10 µM) and a non-specific SIRT2 inhibitor (NAM, 1 mM) as positive controls. In comparison with what occurs in untreated cells, the incubation of THP-1 cells with both inhibitors significantly increased the degree of p53 acetylation by 1.23- and 1.67-fold, respectively. This suggests that NAR and HSP behave as synthetic SIRT2 inhibitors.

### 3.6. NAR and HSP Modulated SIRT2, p21, and Cyclin E1 but Not p53 mRNA Levels in THP-1 Cells

**Figure 8:**
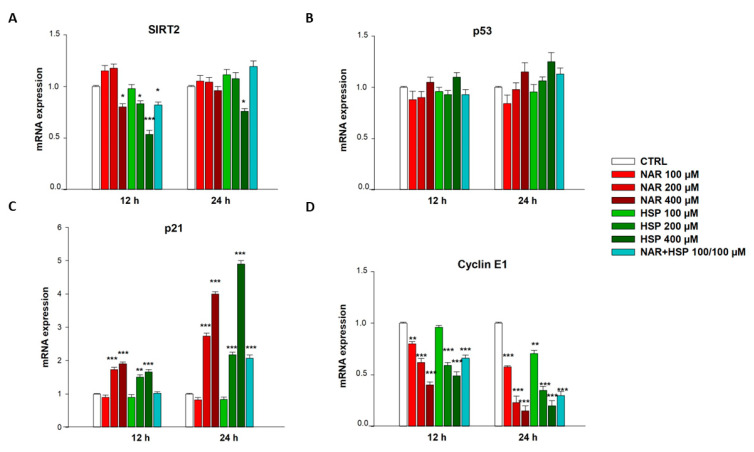
Modulation of SIRT2 (**A**), p53 (**B**), p21 (**C**), and cyclin E1 (**D**) gene expression after treatment with NAR (red bars) and HSP (green bars), as well as their combination (light blue bars), in THP-1 cells for 12 and 24 h. The mRNA levels were quantified by real-time PCR, and their relative quantities were calculated through the 2^–ΔΔCT^ method. Results are expressed as fold change relative to untreated cells. Data are expressed as mean ± SEM of three separate experiments in triplicate (*n* = 9). * *p* < 0.05, ** *p* < 0.01, and *** *p* < 0.001 vs. CTRL.

Besides inhibiting SIRT2 enzymatic activity, NAR and HSP were also able to modulate its gene expression. Indeed, the treatment with both HSP 200 µM and NAR 400 µM for 12 h significantly reduced SIRT2 mRNA levels by 1.25-fold down compared to controls, as well as their equimolar combination of 100 µM concentration, which brought no effects for single flavanones. Of note, the highest tested concentration of HSP (400 µM) significantly lowered SIRT2 mRNA expression after 12 h (1.85-fold down vs. CTRL; Figure 8A). At 24 h of treatment, none of the flavanones was able to alter SIRT2 expression, except for HSP 400 µM, which decreased it by 1.32-fold down vs. CTRL (Figure 8A).

Neither NAR nor HSP altered the gene expression of total p53 for concentrations up to 200 µM at any of the timings tested, maintaining the mRNA quantities at the level detected in untreated cells. However, a non-significant increase in p53 gene expression was observed at 400 µM of NAR and HSP after both 12 h and 24 h (Figure 8B).

Moreover, the treatment of THP-1 cells with NAR and HSP was able to modulate S phase cell cycle-related factors p21 and cyclin E1, already after 12 h of treatment. In particular, neither NAR nor HSP 100 µM modified p21 expression with respect to control cells, whereas this occurred with higher concentrations of both flavanones (200 and 400 µM). In detail, compared to controls, treatment with NAR 200 µM for 12 h increased p21 mRNA levels in THP-1 cells by 1.7-fold and with HSP 200 µM by 1.5-fold. For the concentrations of 400 µM of NAR and HSP, a similar increase in p21 levels (1.9- and 1.7-fold, respectively) was recorded after 12 h. Likewise, this growing trend was maintained after 24 h of treatment with NAR (2.7-fold vs. CTRL) and HSP (2.2-fold vs. CTRL) 200 µM, whereas it was even higher with NAR 400 µM, which significantly raised p21 gene expression by 4.0-fold, and HSP 400 µM by 4.9-fold respect to controls. On this line, after 24 h of incubation, the equimolar combination (100 µM) between the two flavanones increased p21 mRNA levels by 2.1-fold respect control, similarly to HSP 200 µM (Figure 8C).

Contrariwise, cyclin E1 expression was significantly hampered by NAR and HSP already after 12 h of treatment. Indeed, NAR reduced mRNA levels of cyclin E1 by 1.25-, 1.62- and 2.49-fold down at 100, 200, and 400 µM, respectively, whereas HSP lowered cyclin E1 mRNA quantity by 1.70- and 2.04-fold down at 200 and 400 µM, respectively. Their combination decreased by 1.52-fold down cyclin E1 expression after 12 h of incubation. After 24 h of treatment, NAR and HSP significantly reduced cyclin E1 gene expression at 100 µM (2- and 1.43-fold down, respectively), 200 µM (5- and 2.9-fold down, respectively), and 400 µM (6.7- and 5-fold down, respectively). Their association (100/100 µM) brought a result like that of the 200 µM of both NAR and HSP alone (Figure 8D), while the 200 µM combination did not prove any better than that reported (data not shown).

### 3.7. Flavanones Interacted with the Inhibitory Site of SIRT2 Enzyme

To investigate the molecular interactions between the flavanones NAR and HSP with SIRT2, we performed docking simulations using AutoDock4 suite [27], employing crystallographic coordinates of the complexed 4RMG as reference structure [25]. The docking protocol (see Methods) was first validated through self-docking of SIRT2 inhibitor SirReal2, whose best docking pose was in suitable agreement with the experimental structure (RMSD value of 0.5 Å).

**Figure 9:**
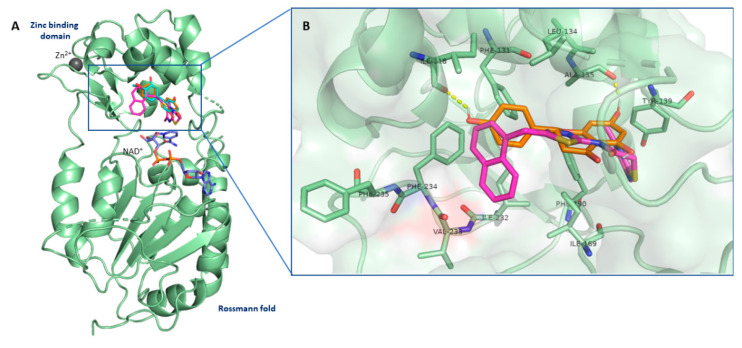
NAR and HSP interacted with the inhibitory site of SIRT2 in silico. (**A**) X-ray pose of SirReal2 (in magenta; PDB code 4RMG) superimposed on the predicted binding pose of NAR (in orange) and HSP (in cyan). (**B**) Interactions of SirReal2 and NAR at selectivity pocket of SIRT2. The crucial residues are shown in stick mode, while hydrogen bonds are shown as yellow dashed lines.

The computational studies suggested that all studied compounds had a similar network of interactions as SirReal2 in the lipophilic pocket of SIRT2, forming crucial contacts within active site residues. The inspection of docking results suggested that the 2-(4-hydroxyphenyl)-substituent of NAR, taken as an example, forms crucial π-T-shaped interaction with Phe131, whereas the chromen-4-one moiety occupies the hydrophobic region close to Tyr139 and Phe190 residues. Moreover, our molecular simulation revealed that NAR, such as HSP, made two hydrogen bond interactions with oxygen atoms of Ile118 and Ala135 backbone. Compared to SirReal2, NAR loses the crucial π-π stacking interaction within the selectivity pocket of SIRT2. This could explain the minor activity of the flavanones (Figure 9).

## 4. Discussion

The main source of dietary flavonoids is *Citrus* fruits, and, due to their presence, these are endowed with well-acknowledged protective activities exploited to defend human health [30,31,32], also in clinical settings [33,34]. Regarding cancer, *Citrus* fruits and their derivatives have been long investigated for their role in this nefarious disease [35,36,37], and flavanones stand out among others [38].

In this work, we started from the assumption that quercetin-derived compounds have been demonstrated to possess SIRT2 inhibition activity [18], and, given the implication of this enzyme in AML [7], we investigated the role of SIRT2 in the anti-leukemic effect of the most common flavanones of *Citrus* spp. Therefore, the first step was to assess the effect of NAR, HSP, and their glycosidic counterparts, NRG and NHP, on the recombinant isolated SIRT2 enzyme. Interestingly, all flavanones were able to inhibit the deacetylase activity of SIRT2, despite to different extent, thus being the first to report these effects.

Previously, we demonstrated how natural products combined may elicit stronger effects than when alone [22]. On this line, we assessed the pair combinations of the four flavanones employing the same abiotic assay as for testing the single compounds. Unlike all the other combinations, mainly exhibiting an antagonistic interaction, NAR and HSP synergistically inhibited SIRT2 activity, reaching a maximum at an equimolar ratio of 100 µM. The synergism between these two flavanones was maintained for equimolar concentrations up to 200 µM and then progressively decreased at higher concentrations.

In the light of the inhibitory effects of the selected flavanones on the SIRT2 recombinant enzyme and that SIRT2 expression was increased in the M5 subtype of AML patients [7], we decided to assess their activity on a well-established cellular model of AML belonging to this subtype, as the human monocytic leukemic THP-1 cells [39]. This is a reliable cell line for functional, preclinical therapeutics, and target identification studies, as well as for investigating monocyte function and differentiation.

Previous reports suggested the capability of many flavonoids as anti-leukemic agents via targeting multiple pathways [40,41]. In our study, we confirmed this assumption, enriching it with further details. Indeed, we demonstrated that NAR and HSP exerted interesting antiproliferative activity in THP-1 cells, whereas the glycosides NRG and NHP showed minimal effect on the proliferation of this cell line, at least at the concentration tested in our study. This outcome is in line with a previous work by Chen and co-workers who claimed that rutinoside in C-7 of flavonoids, the carbohydrate moiety characterizing both NRG and NHP, prevents the induction of antiproliferative activity [42]. Interestingly, these compounds did not exert any toxicity on PBMCs, suggesting a safety profile of flavanones employed in this study.

Given these results, we investigated which type of cell death was elicited by NAR and HSP by assessing whether these compounds can interfere with the progression of THP-1 cells during the cell cycle. This cellular process is governed by checkpoints that are carefully followed by normal cells; nevertheless, if genetic aberrations or abnormalities occur, cells will enter a state of cell cycle arrest [43]. In our experiments, both NAR and HSP were able to block the cell cycle, increasing cells in the S phase at both 48 and 72 h. Notably, the association of the two flavanones retraced what we witnessed in the abiotic assay, where they synergistically acted to inhibit SIRT2 enzymatic activity. Moreover, the hypodiploid population increased in THP-1 cells treated with NAR and HSP 200 and 400 µM, as well as their association (100 µM each), indicating that the elicited block of the cell cycle can lead to apoptotic cell death. The pro-apoptotic activity of 200 µM of NAR in THP-1 cells was also reported by Park and co-workers [44] that linked this property to the activation of caspases and mitochondrial dysfunctions, associated with the inactivation of the PI3K/AKT pathway. Interestingly, we are the first to report the induction of apoptosis elicited by HSP in this cell line. In addition, our results are in line with those of Chen and co-workers [42], who reported that low concentrations of NAR and HSP (i.e., 20, 40, and 80 µM), as well as of their corresponding aglycones, did not exert apoptosis in THP-1 cells, contrariwise to HL-60 ones.

To verify the sirtuin inhibition by flavanones in vitro, we evaluated the level of p53 acetylation, the main cellular target of SIRT2 deacetylase activity [45]. Interestingly, both NAR and HSP 200 and 400 µM hampered p53 deacetylation in a significant manner, like the equimolar association of the two at a lower concentration (100 µM), further corroborating our initial hypothesis of synergism. In this setting, SirReal2, a specific SIRT2 inhibitor, altered p53 acetylation such as NAR and HSP, thus supporting their capability of inhibiting SIRT2 deacetylase activity in vitro. On the other hand, treatment of THP-1 cells with NAM, a non-specific SIRT2 inhibitor, determined a considerable increase in acetylated p53 levels. This is due to a combined effect on more than one sirtuin, which, similarly to the NAM, our flavanones seem to exert [19]. Proving this, the selectivity of NAR and HSP (200 and 400 µM) toward the SIRT2 enzyme seems to straddle that of SirReal2 (10 µM) and NAM (1 mM). However, we saw no change in p53 gene expression for none of the treatments.

Since the enzyme modulation can be achieved by either affecting the activity or the gene expression, we wondered whether the lowered SIRT2 activity could be originated by a decrease in its mRNA levels, in addition to the inhibition of its enzymatic activity. Therefore, when we quantified the total SIRT2 gene levels, we observed an early modulation (12 h) of mRNA expression at the highest tested concentration (400 µM) of NAR and HSP, as well as with their combination at equimolar ratio (100 µM), an effect that was totally abolished at 24 h of treatment, except for HSP 400 µM. This outcome may suggest that cancer cells respond to SIRT2 inhibition with an increase in its gene expression in order to restore its functionality, which is crucial for the survival of AML cells. Notably, HSP 400 µM steadily maintained the suppression of SIRT2 expression, suggesting its potentiality in AML.

During the cell cycle, p53 directly binds DNA to promote the transcription of several factors, among which p21 is the pivotal one [46]. The immediate effect of the p21 increase is the regulation of cyclins such as D and E [47]. In particular, p21 activation leads to inhibition of the cyclin E1, which in turn controls the entry of cells from late G1 to S phase and subsequent S phase arrest. In this regard, we assessed the gene expression of the specific S phase cellular markers p21 and cyclin E1, given the results of cell cycle evaluation. Our results are perfectly in line with what we expected, mirroring initial outcomes from cell cycle analysis. Notably, both NAR and HSP increased p21 expression at different times and concentrations, as well as lowered that of cyclin E1. Interestingly, their combination, also in this case, proved to be effective and comparable to the double of the concentration of the single flavonoids. Therefore, we presumed that THP-1 cell cycle blockage by NAR or HSP may be mediated by p53 activity, but we do not exclude that other p53-independent signaling pathways, closely related to SIRT2, may be involved in the S arrest, as previously suggested [48].

Finally, to understand how NAR and HSP interact with SIRT2, we investigated the putative binding mechanism of these two flavanones by computational techniques. SIRT2 structure was fully deciphered in 2001 by Finnin and co-workers [49] who revealed that SIRT2 possesses a catalytic core domain with NAD^+^-binding capacity as well as N- and C-terminal extensions. The catalytic core contains a variant of the Rossmann fold and a small domain consisting of helical and zinc-binding modules. The abovementioned domains are separated by a large lipophilic area that represents a wide groove containing an active site where deacetylation of substrates occurs. The catalytic groove accommodates various inhibitors from natural and synthetic sources [29,50]. The X-ray crystal structure of the 2-(4,6-dimethyl-pyrimidin-2-ylsulfanyl)-N-(5-naphthalen-1-ylmethyl-thiazol-2-yl)-acetamide (SirReal2) in complex with SIRT2 (PDB code 4RMG) [25] revealed that the inhibitor occupies the “selectivity pocket” shaped by Ile93, Ala135, Leu138, Pro140, Phe143, Leu206, and Ile213. In this hydrophobic pocket, the dimethylmercaptopyrimidine moiety of SirReal2 establishes π-π stacking interactions with Tyr139 and Phe190. Moreover, the naphthyl group of SirReal2 protrudes into the acetyl-lysine channel that is considered the substrate binding site comprising various hydrophobic residues (Phe131, Leu134, Ile 169, Ile232, Val233, Phe234). In detail, the inhibitor engages π-T-shaped interactions with Phe131 and Phe234.

## 5. Conclusions

Hematological malignancies continue to represent a significant challenge, being frequently depicted as incurable diseases. Therefore, urgent development of novel therapeutic agents is needed to overcome the failure of standard therapies and to improve the patients’ survival rate. Some plant-derived products, such as flavonoids, have gained great interest due to their pharmacological potential. Among these, we demonstrated that NAR and HSP, the two most common flavanones of *Citrus* fruits, can exert anti-leukemic effects on the human leukemic monocytic cell line THP-1 through growth inhibition via the arrest of cell cycle progression. Interestingly, these mechanisms appear to be linked to the inhibition of SIRT2, a known proliferation marker in relapsing AML, which makes flavonoids attractive candidates for the management of this pathology. In addition, we showed that NAR and HSP can reciprocally either enhance their antiproliferative effects, thus providing further new possibilities for studying the combined anti-leukemic activity of natural products.

## Figures and Tables

**Table 1:** Oligonucleotide primer sequences used for the quantitative real-time PCR analysis.

**Gene**	**GenBank Accession Number**	**Primer Sequence**
SIRT2	NM_012237.4	Forward: 5′-TTCAAGCCAACCATCTGT-3′ Reverse: 5′-GTATCTATGTTCTGCGTGTAG-3′
TP53	NM_000546.6	Forward: 5′-GTGTGGAGTATTTGGATGAC-3′ Reverse: 5′-ATGTAGTTGTAGTGGATGGT-3′
CDKN1	NM_000389.5	Forward: 5′-TTCTCCACCTAGACTGTAA-3′ Reverse: 5′-GCACCTGCTGTATATTCA-3′
CCNE1	NM_001238.4	Forward: 5′-GGAAGAGGAAGGCAAACGTGA-3′ Reverse: 5′-TCGATTTTGGCCATTTCTTCAT-3′
ACTB	NM_001101.5	Forward: 5′-TTGTTACAGGAAGTCCCTTGCC-3′ Reverse: 5′-ATGCTATCACCTCCCCTGTGTG-3′

## Data Availability

Not applicable.
